# The Terneuzen Birth Cohort: BMI Change between 2 and 6 Years Is Most Predictive of Adult Cardiometabolic Risk

**DOI:** 10.1371/journal.pone.0013966

**Published:** 2010-11-12

**Authors:** Marlou L. A. de Kroon, Carry M. Renders, Jacobus P. van Wouwe, Stef van Buuren, Remy A. Hirasing

**Affiliations:** 1 Department of Public and Occupational Health, EMGO Institute for Health and Care Research, VU University Medical Centre, Amsterdam, The Netherlands; 2 Prevention and Public Health, Department of Health Sciences, and EMGO Institute for Health and Care Research, VU University, Amsterdam, The Netherlands; 3 Netherlands Organisation for Applied Scientific Research, TNO Quality of Life, Prevention and Health Care, Leiden, The Netherlands; 4 Department of Methodology and Statistics, FSS, University of Utrecht, Utrecht, The Netherlands; Aga Khan University, Pakistan

## Abstract

**Background:**

We recently reported the age interval 2–6y being the earliest and most critical for adult overweight. We now aim to determine which age intervals are predictive of cardiometabolic risk at young adulthood.

**Methods and Findings:**

We analyzed data from 642 18–28 years olds from the Terneuzen Birth Cohort. Individual BMI SDS trajectories were fitted by a piecewise linear model. By multiple regression analyses relationships were assessed between subsequent conditional BMI SDS changes and components of the metabolic syndrome (MetS), skinfold thickness and hsCRP at young adulthood. Results were adjusted for gender and age, and other confounders. Gender was studied as an effect modifier. All BMI SDS changes throughout childhood were related to waist circumference and skinfold thickness. No other significant relationship was found before the age of 2 years, except between the BMI SDS change 0–1y and hsCRP. Fasting blood glucose was not predicted by any BMI SDS change. BMI SDS change 2–6y was strongly related to most outcome variables, especially to waist circumference (ß 0.47, SE 0.02), systolic and diastolic blood pressure (ß 0.20 SE 0.04 and ß 0.19 SE 0.03), and hsCRP (ß 0.16 SE 0.04). The BMI SDS change 10–18y was most strongly related to HDL cholesterol (ß -0.10, SE 0.03), and triglycerides (ß 0.21, SE 0.03). To a lesser degree, the BMI SDS change 6–10y was related to most outcome variables. BMI SDS changes 2–6y and 10–18y were significantly related to MetS: the OR was respectively 3.39 (95%CI 2.33–4.94) and 2.84 (95%CI 1.94–4.15).

**Conclusion:**

BMI SDS changes from 2y onwards were related to cardiometabolic risk at young adulthood, the age interval 2–6y being the most predictive. Monitoring and stabilizing the BMI SDS of children as young as 2–6y may not only reverse the progression towards adult overweight, but it may also safeguard cardiometabolic status.

## Introduction

The high prevalence of overweight and obesity in children is worrisome as among others it is related to cardiometabolic risk even at young ages. In childhood, not only the BMI status itself, but particularly the BMI increase, is strongly related to cardiometabolic risk at adulthood [1]. As an increase in BMI SDS implies a more than normal increase in the BMI with age as such, an increase in BMI SDS during childhood might explain the high cardiometabolic risk even in adults with a normal BMI [2], [3]. In the Terneuzen Birth Cohort, we found the age interval from 2 to 6 years to be the earliest and most critical growth period for adult overweight [4]. As overweight reflects total body mass and not only body fat mass, we question whether this age interval is also most predictive of cardiometabolic risk.

Several studies have addressed the relation between BMI increase in childhood and cardiometabolic risk at adulthood [5]–[9]. Longitudinal data from birth into adulthood are needed to estimate the relative contribution of subsequent changes in BMI SDS to cardiometabolic risk. Only a few studies have valid longitudinal data to study these relationships [5]–[7]. Study results differ with respect to which age interval is most predictive. One study has assessed adolescence to be the only sensitive period for developing visceral fat at adulthood [6]. Others have shown that the BMI increase from 2 years onwards is associated with cardiometabolic risk [5]. Yet another study did not find a critical age interval: the weight increase from birth onwards had an evenly spread influence on adult fat patterns [7]. Height and weight data collected from birth up until young adulthood are at our disposal. We aim to find whether there is a most critical age interval predictive of cardiometabolic risk at young adulthood.

## Methods

### Population and Study Design

#### Ethics statement

The study protocol was approved by the Medical Ethics Committee of the VU University Medical Centre Amsterdam, and written informed consent was obtained from all the participants.

#### Population

The Terneuzen Birth Cohort consists of all 2,604 children born between 1977–1986 in the city of Terneuzen, the Netherlands [3]. Data for height and weight from 1,701 subjects as registered according to a standard protocol by the Municipal Health Services were available from birth until adolescence. These subjects were invited to participate in a follow-up study that included a physical examination, blood tests and a questionnaire to collect sociodemographic characteristics and data about cigarette smoking and their mothers' actual BMI. Of these 1,701 subjects, 577 could not be traced, 362 completely refused to participate, and 120 refused to participate in blood drawing. Therefore the analyses were restricted to the remaining 642 subjects. The males and females in this follow-up study do not differ from the original cohort regarding baseline characteristics, i.e. age, birth weight, BMI SDS at birth**,** age of the mother, parity, and breastfeeding. The only difference was the difference for gender itself (41% males vs. 51% in the original cohort, *p*<0.05). We used BMI values (kg/m^2^) as the measure for (over)weight, converted to age-specific standard deviation scores (BMI SDS) based on Dutch reference data [10] most comparable to our study population.

#### Physical examination and blood tests

Physical examinations were performed by two assistants who received standardized training at the Municipal Health Services in Terneuzen (GGD Zeeland). Waist circumference was measured mid-way between the lower side of the lowest rib and the upper side of the pelvis, on bare skin, after a normal expiration, and with muscles relaxed. Blood pressure (BP) was measured twice (with a 5-minute rest interval) on the left upper arm with the Omron 5–1, which is a fully automatic blood pressure monitor. The mean values of systolic blood pressure (SBP) and diastolic blood pressure (DBP) were used as outcomes. Triceps, biceps, subscapular and suprailiacal skinfolds were measured three times to the nearest 0.1 mm with a Holtain skinfold caliper (Holtain Ltd, Crosswell, United Kingdom) on the left side of the body. Skinfold thickness was defined as the sum of the mean values of the three measurements of every skinfold.

Fasting venous blood samples were drawn in the clinical chemistry laboratory of the Community Hospital at Terneuzen. After centrifugation (10 minutes 1500xG), plasma was analyzed with a routine clinical chemical analyzer, Synchron LX20PRO (Beckman Coulter Inc., USA). Glucose, HDL cholesterol, triglycerides and high-sensitivity C-reactive protein (hsCRP) were measured. Detailed information about the anthropometric measurements and the blood tests is described elsewhere [3]. An external quality control was performed [11]–[13]. Metabolic syndrome, a progressive disorder and a useful tool for the long-term risk assessment of cardiometabolic diseases, was defined using the NCEP-ATPIII definition [14].

Based on reported associations between rising BMI SDS and cardiometabolic risk and characteristics of human growth, we divided the age scale *a priori* into the following intervals: birth-1 year [15], [16], 1–2 years [17], [18], 2–6 years [19], [20], 6–10 years, and 10–18 years [21], [22].

We used narrow age intervals between birth and 2 years because of the rapid changes in the BMI during infancy: during the first year of life the BMI mostly increases and during the second year it decreases [16]. In addition, the BMI at 1 year of age is strongly associated with the BMI at 7 years at age [15]. The age limit at two years was chosen because it has been shown that a rapid weight gain in the first two years of life is associated with adolescent overweight [18], and that adults with impaired glucose tolerance or diabetes have an accelerated BMI increase from two years onward [17]. The age of 6 years tallies with the approximate age of adiposity rebound [16], [19], and the onset of adrenarche in children [20]. The upper limit in the interval 6–10 years (y) was chosen since most children go into puberty after 10 years of age. The upper limit of 18 years was chosen because it marks the start of adulthood and the cessation of height growth.

#### Statistical analyses

The average number of BMI data points per subject between 0–18 years is 21. The means (and SD) of the numbers of BMI measurements in the age intervals birth-1y, 1-2y, 2-6y, 6-10y and 10-18y are respectively 12.8 (2.1), 1.8 (0.7), 2.7 (0.9), 1.8 (0.9) and 1.9 (0.7). Individual BMI SDS trajectories were fitted by a piecewise linear model otherwise known as the broken-stick model [23], which has been described in more detail in a previous manuscript [9], [24]. For each subject, this model approximates the observed BMI SDS trajectory by a series of straight lines that connect to each other at the break ages. The expected value of BMI SDS at a break age is called the *status score*. The change between the status score at the start and the end of the various age intervals is called the *change score.* The S Plus 8.0 function bs() was used to perform these analyses. For further analyses we have used the status scores from the multilevel analysis instead of the raw data.

To assess the relative contribution of the respective age intervals, change scores in the age intervals were regressed on the BMI SDS at birth and all previous change scores. When relationships between change scores are nonlinear, quadratic terms of the independent variables were included in these regression calculations. By expressing residuals as BMI SDS changes, uncorrelated independent variables describing BMI SDS changes, *conditional change scores,* were obtained, and regression to the mean was taken into account [25]. Associations between BMI SDS changes in early life and adult outcomes were examined using linear and logistic regression analysis, in which the BMI SDS at birth and subsequent conditional change scores were all included in one model. This implies that if a conditional change score turned out to be a significant predictor of the outcome variable at adulthood, the change score is a significant predictor of the outcome variable, irrespective of the BMI SDS at birth and preceding change scores. Age, gender, exclusive breastfeeding (<90 vs. ≥90 days), cigarette smoking (none vs. ≥1 cigarette a day) of the subject, and parity and BMI of the mother were studied as possible confounders. Effect modification for gender was tested by including the interaction between gender and change scores (at a type 1 error rate of 0.05). Non-linearity was tested by adding quadratic terms of subsequent conditional BMI SDS scores to the models (at a type 1 error rate of 0.05). Standardized regression coefficients were calculated to estimate the impact of a unit standard deviation in the predictor.

The outcome variables were the components of the metabolic syndrome, skinfold thickness and hsCRP value. The age of the subjects varied between 18 and 28 years. The residuals of the blood concentrations and SBP and DBP showed a skewed distribution. These variables were log-transformed so that the distribution is closer to normal. The regression coefficients and outcome variables were standardized so that the effects on different outcomes can be compared quantitatively. Results were adjusted for gender and age. As far as indications existed for confounding by parity, exclusive breastfeeding, smoking behavior, and BMI of the mother (*p*<0.10), the outcomes were also adjusted for these variables.

For the models with the highest (adjusted) explained variance (i.e. ≥0.25), the effects of the change scores on the levels of the outcome variables at young adulthood were quantified. The change in the levels of these outcomes was calculated for a hypothetical increase in the conditional change score from 0 to 1 SDS for each age interval.

## Results

Characteristics of the study population (n = 642) and the results of the anthropometric measurements and blood tests are shown in Table 1. Significant differences between adult males and females are found for the levels of waist circumference, skinfold thickness, HDL cholesterol, hsCRP, fasting glucose and systolic blood pressure.

**Table 1:** Characteristics of the study population (n = 642) and the outcomes of the anthropometric measurements and blood tests by gender.

	Males			Females		
Characteristics	n	mean	SD	n	mean	SD
age at young adulthood (y)	265	23.10	2.92	377	23.04	2.94
BMI at young adulthood (kg/m^2^)	265	23.06	3.40	377	23.58	3.94
BMI mother (kg/m^2^)	218	24.9	3.88	323	25.72	4.41
BMI father (kg/m^2^)	210	26.05	2.95	285	26.31	3.08
waist circumference (cm)*	265	84.31	9.75	377	79.13	10.19
skinfold thickness (mm)*	264	44.85	22.00	372	67.51	26.16
HDL cholesterol (mmol/L)*	265	1.25	0.92	377	1.48	0.33
triglycerides (mmol/L)	265	0.95	0.61	377	0.95	0.53
hsCRP (mg/L)*	264	2.42	3.60	364	3.24	3.74
fasting glucose (mmol/L)*	265	5.23	0.55	377	5.00	0.02
systolic blood pressure (mmHg)*	265	135.01	13.15	377	121.94	11.74
diastolic blood pressure (mmHg)	265	76.05	8.76	377	76.05	9.01
	**n**	**%**		**n**	**%**	
parity (% firstborn)	265	58.1		377	61.8	
breastfeeding (≥90 days)	265	24.9		377	25.2	
smoking behaviour	265	24.2		377	23.9	
metabolic syndrome	265	6.4		377	8.2	

*statistically significant difference between males and females (p<0.05).

Table 2 shows the standardized regression coefficients for cardiometabolic risk factors at young adulthood as a result of the multiple linear regression analyses. BMI SDS at birth and the conditional change scores were modeled as independent variables. No evidence was found for confounding by exclusive breastfeeding and smoking behavior of the subject, or parity and BMI of the mother, so these variables were not included in the final models. The quadratic term of the conditional change score did not appear to be significantly related to the outcome variables (*p*>0.05) for any of the age intervals.

**Table 2:** Health outcomes at young adulthood by multiple and logistic regression analyses in models including BMI SDS at birth and the conditional change scores.

	Standardized regression coefficients and 95% CI																		
Outcome variables (in SDS)	BMI SDS at birth			change scorebirth - 1y B			change score1-2y B			change score2-6y B			change score6-10y B			change score10-18y B			Adjusted explained variance
waist circumference	**0.10**	**0.04–0.16**	**	**0.24**	**0.18–0.30**	**	**0.12**	**0.08–0.16**	**	**0.47**	**0.43–0.51**	**	**0.23**	**0.17–0.29**	**	**0.36**	**0.32–0.40**	**	0.60
skinfold thickness	**0.07**	**0.01–0.13**	**	**0.20**	**0.14–0.26**	**	**0.09**	**0.03–0.15**	**	**0.38**	**0.32–0.44**	**	**0.24**	**0.18–0.30**	**	**0.33**	**0.29–0.37**	**	0.56
systolic blood pressure^A^	0.02	−0.04–0.08		0.01	−0.05–0.07		0.00	−0.06–0.06		**0.19**	**0.13–0.25**	**	**0.11**	**0.05–0.17**	**	**0.11**	**0.05–0.17**	**	0.28
diastolic blood pressure^A^	0.02	−0.06–0.10		0.04	−0.04–0.12		0.01	−0.07–0.09		**0.20**	**0.12–0.28**	**	**0.10**	**0.02–0.18**	*	0.04	−0.02–0.10		0.06
HDL cholesterol^A^	−0.01	−0.09–0.07		−0.07	−0.15–0.01		−0.05	−0.13–0.03		−**0.08**	−**0.16**–**0.00**	*	**0.09**	**0.01–0.17**	*	**−0.10**	**−0.16–−0.04**	**	0.18
triglycerides^A^	0.00	−0.08– 0.08		0.00	−0.08–−0.08		−0.06	−0.14–0.02		**0.18**	**0.10–0.26**	**	**0.08**	**0.00–0.16**	*	**0.21**	**0.15–0.27**	**	0.09
fasting glucose^A^	−0.03	−0.11–0.05		0.02	−0.06–0.10		−0.06	−0.14–0.02		0.06	−0.02–0.14		0.00	−0.08–0.08		0.05	−0.01–0.11		0.05
hsCRP^A^	−0.04	−0.12–0.04		**0.10**	**0.02–0.18**	*	0.02	−0.06–0.10		**0.16**	**0.08–0.24**	**	**0.09**	**0.01–0.17**	*	**0.15**	**0.09–0.21**	**	0.27
	Odds ratios and 95%CI																		
MetS	1.31	0.88–1.93		1.30	0.88–1.93		0.95	0.64–1.40		**3.39**	**2.33–4.94**	**	1.30	0.93–1.82		**2.84**	**1.94–4.15**	**	

All analyses are adjusted for age and gender.

*0.002<p<0.05,

**p<0.002 (statistically significant relations are printed in bold; *p*<0.05).

A Log transformed variables.

B The independent variables between birth and 18y are conditional measures. i.e, they are regressed on the BMI SDS at birth and previous change scores, so the BMI SDS changes are uncorrelated. The formula to obtain the conditional change scores is X_res n_  =  Y - α - β ^.^ Z_0_ - γ_(n-(n-1))_^.^_._ X_res (n-(n-1))_ - γ _(n-(n-2))_ X_res (n-(n-2))_ - ...... - γ _(n-1)._ X_res (n-1)_, where Z_0_ equals BMI SDS at birth, X_res n_ conditional change scores, and Y the outcome variable. If statistically significant the quadratic term of X_res n_ was added to the formula.

The explained variance is highest for the outcomes of the two anthropometric measurements, waist circumference and skinfold thickness, followed by systolic blood pressure and the hsCRP level. All conditional change scores from birth onwards show a significant relationship with waist circumference and skinfold thickness at young adulthood. The conditional change score 2-6y is the only significant predictor for all outcome variables, with the exception for fasting glucose for which no significant relation was found for any age interval. Moreover, the conditional change score 2-6y is most predictive of the outcome variables, with the exception for triglycerides and HDL cholesterol. It is noteworthy that the regression coefficient of 6-10y was positive for HDL cholesterol, whereas for other age intervals, the regression coefficient was negative. Apart from the anthropometric outcomes, the only other outcome that was predicted by a BMI increase before the age of 2 years was hsCRP, i.e. by change score 0-1y. The odds ratios of MetS at young adulthood are also shown. Significant odds ratios were found for the conditional change scores 2-6y and 10-18y. The age interval 2-6y and after that 10-18y are most strongly related to the prevalence of MetS (OR was 3.39 and 2.84 respectively).

**Figure 1:**
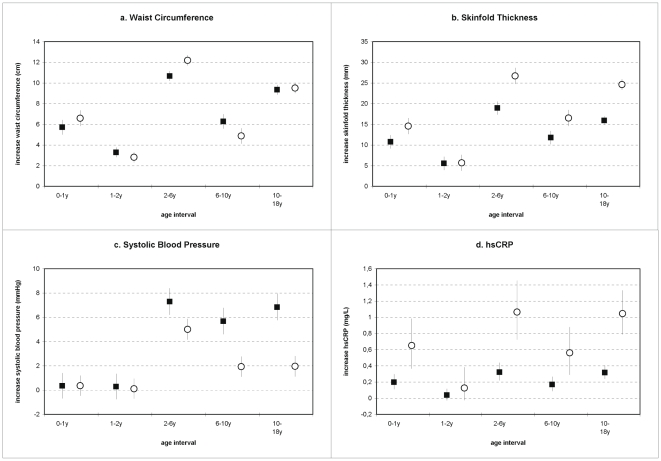
Association between the conditional change score from 0 to +1 SDS for the respective age intervals and the actual change (+/− 1 SE) of several outcome variables. Outcome variables are respectively a) waist circumference (cm), b) skinfold thickness (mm), c) systolic blood pressure (mm Hg) and d) hsCRP (mG/L) at 23y (males: black squares, females: white circles)

Figure 1 shows associations between the conditional change score from 0 to +1 SDS for the respective age intervals for the models with the highest adjusted explained variance (>0.25) and the actual changes in the values of the outcome variables. The change in these outcome variables is highest for the age interval 2-6y. This figure also shows differences between males and females.

## Discussion

Our aim was to assess the relationship of change scores during childhood (BMI SDS changes) with cardiometabolic risk at young adulthood. All BMI changes, including those before the age of 2 years, were significantly related with waist circumference and skinfold thickness at adult age. However, the association between BMI changes and other cardiometabolic risk factors at adulthood, except for hsCRP, only became apparent from the age of 2y onwards. The change scores 2-6y and 10-18y were most predictive of the outcome variables (Table 2). An unexpected finding was that the relationship between the change score 6-10y and HDL cholesterol at young adulthood was positive (*p* = 0.004). The change scores 2-6y and 10-18y were associated with high risk for metabolic syndrome, with the change score 2-6y having the highest odds-ratio (3.39, 95%CI 2.33–4.94).

Our results are highly similar to the findings of the New Delhi Birth Cohort, even though the studied populations differ to a great extent with respect to ethnicity and welfare [5]. In both cohorts the BMI changes from 2 years onwards are associated with cardiometabolic risk. Due to the chosen study design, we were able to distinguish the impact of relatively small age intervals.

For the first two years of life, the available evidence to date points in different directions [7], [26]–[28]. We found, in line with the New Delhi Birth Cohort [5], the BMI SDS increase between birth and the age of 2 years to be related only to waist circumference and skinfold thickness at young adulthood, whereas for all other identical outcomes in both cohort studies no significant relationships were found. In addition, we found that the BMI SDS increase between birth and 1y is also related to hsCRP, a strong predictor of cardiometabolic risk [29], which was not included in the Delhi Birth Cohort study. This would suggest that the hsCRP is more sensitive to BMI SDS increase between 0-1y than other studied outcomes. Generally, the age interval 1-2y showed even weaker associations than the age interval 0-1y. What happens during the age interval 1-2y seems to have very little effect on later cardiometabolic risk. We have no clear explanation for this finding. The way the fat mass expands at different ages during childhood, either by increasing the number of adipocytes or by increasing fat cell volume[30], [31], and/or the development of motor skills, may play a role.

Another study found that the age interval 1 to 5 years predicts systolic blood pressure whereas before 1 year of age no relationship was found [28]. This finding also seems in line with our results as the age intervals 1-5y and 2-6y largely overlap. In contrast, some other studies have found a significant relationship of growth before the age of one or two years with blood pressure, HDL cholesterol or triglycerides [26], [27]. These different findings might be due to specific characteristics of the populations studied, such as small for gestational age at birth or short stature at adulthood [26], [27]. Further research, addressing the relationships between population characteristics, such as prematurity and dysmaturity, and growth parameters during the first two years of life, might clarify the differences between some of these study results.

The positive regression weight between the change score 6-10y and HDL level at young adulthood (*p* = 0.004) is not in accordance with the expected negative regression weights of all other age intervals. An explanation for this finding might be the supposedly protective effect of subcutaneous fat against cardiometabolic risk [32], which during late childhood contributes more to the BMI increase than visceral fat [6]. Further research is needed to investigate this hypothesis. The protective effect of subcutaneous fat might also partly explain that the association between the BMI SDS change in this age interval and the outcome variables is generally weaker than in the age intervals 2-6y and 10-18y.

As shown in Figure 1, an increase in the conditional change scores from 0 to +1 BMI SDS is associated with substantial increases in waist circumference, skinfold thickness, systolic blood pressure and hsCRP at young adulthood. Consistent with other studies [14], [33]–[35], the levels of waist circumference, skinfold thickness, systolic blood pressure and hsCRP for males and females differ (Table 1). As no significant interaction between change scores and gender has been found, the different increases for males and females are due to the different levels for waist circumference, skinfold thickness, systolic blood pressure and hsCRP for males and females. In relative terms, the found increases in these outcome variables are approximately the same for both sexes.

Our study has some limitations. The age intervals were chosen on substantive grounds. However, it is noteworthy that this choice might have influenced the study results. By combining subsequent age intervals, the relationship with the outcome variable will be an average of the relationships of these age intervals. Also, increasing the width of an age interval generally increases the chance that a significant relationship will be found and vice versa. As in most birth cohorts, there was a loss to follow-up [5], [6]. However, selection bias is very unlikely. First, the males and females who participated were representative with regard to the baseline characteristics of the original cohort [3], and second, for this within-sample analysis, there is no reason to assume that a difference exists between those included and not included in the study with regard to the relationships of the changes in BMI status at childhood with the studied outcomes at adulthood. The strength of our study is that it is population-based, with an average of 21 measurements between birth and young adulthood. Also, all height and weight measurements throughout childhood were recorded prospectively and the measurements in adulthood have been performed according to a protocol by specially trained personnel.

Notwithstanding the fact that several findings in our study are reason for further research or give rise to new hypotheses, our study shows that BMI SDS changes from 2 to 18 years are related to increased cardiometabolic risk at young adulthood, the age interval 2-6y being the most predictive. Along with the highest odds ratio on MetS for this age interval, our findings suggest that preventing BMI SDS increase during this age interval has the potential to prevent cardiometabolic disease at adulthood. Monitoring and stabilizing the BMI SDS of children as young as 2-6y may not only reverse the development towards adult overweight [4], it may also safeguard cardiometabolic status.
